# Wirtschaftspolitische Implikationen der COVID-19-Pandemie im Luftverkehr

**DOI:** 10.1007/s10273-021-2851-3

**Published:** 2021-02-20

**Authors:** Janina Scheelhaase, David Ennen, Benjamin Frieske, Klaus Lütjens, Sven Maertens, Florian Wozny

**Affiliations:** 1 DLR - Institut für Flughafenwesen und Luftverkehr, Linder Höhe, 51147 Köln-Porz, Deutschland; 2 DLR - Institut für Fahrzeugkonzepte, Fahrzeugsysteme u.a., Pfaffenwaldring 38-40, 70569 Stuttgart, Deutschland; 3 LY - Lufttransportbetrieb und -infrastrukturen, DLR - Institut für Lufttransportsysteme, Blohmstraße 20, 21079 Hamburg, Deutschland; 4 FW - Luftverkehrsforschung, DLR - Institut für Flughafenwesen und Luftverkehr, Linder Höhe, 51147 Köln-Porz, Deutschland

**Keywords:** L93, L98, G32

## Abstract

Der Luftverkehr ist durch die Maßnahmen gegen die COVID-19-Pandemie besonders stark betroffen. Der Verkehrsrückgang betrug 2020 zum Teil mehr als 90 %. Da die Branche von großer Bedeutung ist, sind besondere staatliche Unterstützungsmaßnahmen sinnvoll, wenn die Pandemie vorbei ist und neues Wachstum einsetzt. Dabei sollten jedoch nicht nur bestimmte Unternehmen unterstützt werden, sondern die Hilfsmaßnahmen sollten auch an bestimmte Kriterien gebunden sein.

## Abstract (en)

global air transport passenger traffic has been particularly affected by measures to combat COVID-19. Since February 2020, both the number of global passengers and passenger flight kilometers has decreased worldwide — in Western Europe by about 80 % by the end of 2020. These developments have caused financial losses for both airlines and airports worldwide, critically threatening the existence of a large number of these companies. As the industry is of great economic importance, special government support measures could contribute to the economic recovery of the aviation industry. However, not only should certain companies be supported, but the support measures should be linked to defined and transparent criteria.

Die – noch andauernde – COVID-19-Pandemie hat zu einem beispiellosen, bis heute (Februar 2021) anhaltenden Rückgang des weltweiten Passagierluftverkehrs geführt, ausgelöst durch eine Kombination aus Nachfrage- und Angebotsschock. Nachfrageseitig sind durch weltweite Regelungen zur physischen Distanz, Einreiseverbote und Grenzschließungen die wesentlichen Gründe für Flugreisen, also Geschäftstermine, private Besuche und Tourismus, überwiegend weggefallen. Hinzu kommt die Verunsicherung vieler Menschen über die Ansteckungsgefahr während einer Reise. Schließlich führt die Pandemie trotz staatlicher Hilfsprogramme bei vielen Menschen zu Einkommensverlusten. Auf der Angebotsseite wurde diese Entwicklung durch behördlich angeordnete Angebotsreduzierungen flankiert, etwa in Form von Einflugverboten. Zudem wirken hier Kosten, die Flughäfen und Fluggesellschaften durch zusätzliche Hygienemaßnahmen entstehen. Schließlich sind die Fluggesellschaften durch die bisher genannten nachfrage- und angebotsseitigen Effekte gezwungen, Streckennetze und Angebotsfrequenzen deutlich zu reduzieren, was wiederum die Attraktivität des Transportangebots und damit die Luftverkehrsnachfrage weiter reduzieren kann. Somit sind zumindest vorübergehend wesentliche Treiber des Luftverkehrswachstums weggefallen und haben sich sogar ins Gegenteil verkehrt. Für die Luftverkehrsindustrie ist dies eine existenzbedrohende Krise. Kaum ein Unternehmen ist in der Lage, einen solchen Zustand über Monate oder gar Jahre aus eigener Kraft aufzufangen.

**Dr. Janina Scheelhaase** leitet die Abteilung Luftverkehrsökonomie am Institut für Flughafenwesen und Luftverkehr des Deutschen Zentrums für Luft- und Raumfahrt (DLR) in Köln.

**Dr. David Ennen** ist dort wissenschaftlicher Mitarbeiter.

**Benjamin Frieske** ist wissenschaftlicher Mitarbeiter am Institut für Fahrzeugkonzepte, Fahrzeugsysteme und Technologiebewertung des DLR in Stuttgart.

**Klaus Lütjens** leitet die Abteilung Lufttransportbetrieb und -infrastrukturen an der Einrichtung Lufttransportsysteme des DLR in Hamburg.

**Dr. Sven Maertens** und **Dr. Florian Wozny** sind wissenschaftliche Mitarbeiter am Institut für Flughafenwesen und Luftverkehr des DLR in Köln.

Für Gesellschaft und Staat stellt sich die Frage, ob und wie Unternehmen in dieser Krise geholfen werden kann und soll. Aus der Erfahrung von früheren Krisen verfügt Deutschland über das Instrument der Kurzarbeit, das insbesondere zur Beschäftigungssicherung dient, aber auch den Unternehmen, ohne Kündigungen aussprechen zu müssen, einen Großteil der Personalkosten abnimmt. Darüber hinaus stehen über die Kreditanstalt für Wiederaufbau (KfW) für alle Branchen weitere Hilfsinstrumente in Form von Zuschüssen und Krediten zur Verfügung. Da die Luftverkehrsindustrie in besonderer Weise von dieser Krise betroffen ist und darüber hinaus von der Politik ein strategisches Interesse an dieser Branche besteht, wurden zusätzliche Maßnahmen, wie eine Beteiligung des Staates als Anteilseigner und größere Kreditpakete, in Erwägung gezogen, und im Fall der Deutschen Lufthansa auch schon umgesetzt.

### Effekte der Corona-Pandemie auf den internationalen, europäischen und nationalen Luftverkehr

Die International Air Transport Association (IATA, 2019), ein Interessensverband von knapp 300 Airlines, ging in ihrem ökonomischen Jahresbericht vom 11. Dezember 2019 von einer Steigerung der Passagiermenge, der Verkehrsleistung (Passagierkilometer, *revenue passenger kilometers*, kurz: RPK) und der Ticketerlöse im weltweiten Passagierluftverkehr von durchschnittlich je rund 4 % im Jahr 2020 aus. Dabei wurde für Lateinamerika (+4,3 %) und Asien-Pazifik (+4,8 %) ein über- und für Afrika, Europa und Nordamerika (je +3,8 %) sowie den Mittleren Osten (+2,5 %) ein unterdurchschnittliches Wachstum der Verkehrsleistung (RPK) erwartet.

Im Januar 2020 wurden die Auswirkungen des grassierenden Virus auf den Luftverkehr erstmals sichtbar, als die chinesischen Behörden die Stadt Wuhan unter Quarantäne setzten und eine Einstellung unter anderem des Luftverkehrs aus Wuhan ab dem 23. Januar anordneten (ch-aviation, 2020a). Bereits am 27. Januar folgte ein Verbot sämtlicher Auslandsreisen chinesischer Reisegruppen und Pau-

Abbildung 1
**Globale Luftverkehrsentwicklung im Vorjahresvergleich**

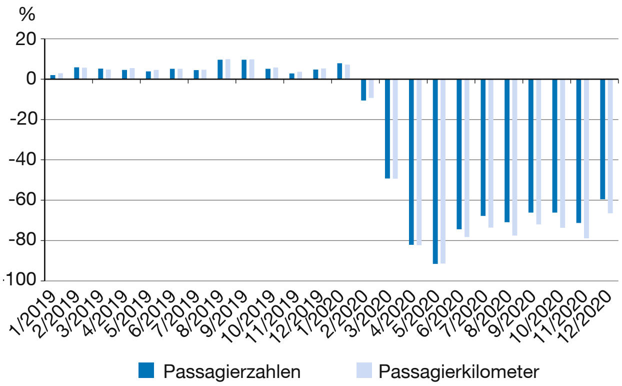

Quelle: Sabre (2021), ab Oktober 2020 vorläufige Daten.

Tabelle 1
**Passagierrückgänge im weltweiten Luftverkehr nach Abflugregion (stärkster Rückgang blau unterlegt)**

| Abflugregion | Marktgröße (Passagierzahl 2019) | 2019 | Jan. 2020 | Feb. 2020 | Mrz. 2020 | Apr. 2020 | Mai 2020 | Jun. 2020 | Jul. 2020 | Aug. 2020 | Sep. 2020 | Okt. 2020 | Nov. 2020 | Dez. 2020 | Mrz.-Dez. 2020 |
|---|---|---|---|---|---|---|---|---|---|---|---|---|---|---|---|
| | | Veränderung im Vergleich zur Vorjahresperiode (in %) | | | | | | | | | | | | | |
| Nordamerika | 1.100.119.272 | 4,0 | 5,8 | 6,2 | -50,4 | -91,3 | -88,7 | -79,0 | -70,6 | -69,9 | -62,4 | -78,2 | -71,9 | -50,2 | -71,2 |
| Fernost | 1.000.838.858 | 8,6 | 10,3 | -49,0 | -65,8 | -69,6 | -89,3 | -80,6 | -57,2 | -54,8 | -52,7 | -37,7 | -52,8 | -51,3 | -58,4 |
| Westeuropa | 914.587.697 | 7,7 | 1,1 | -1,8 | -50,8 | -89,3 | -94,6 | -89,6 | -72,5 | -77,5 | -74,5 | -79,2 | -89,1 | -76,8 | -79,2 |
| Südostasien | 408.284.637 | 0,7 | 16,0 | -8,7 | -42,9 | -70,4 | -96,6 | -91,6 | -60,7 | -76,0 | -75,2 | -63,9 | -74,9 | -71,1 | -70,1 |
| Osteuropa | 301.012.257 | 6,2 | 11,2 | 3,1 | -36,1 | -85,5 | -93,6 | -78,8 | -59,4 | -64,1 | -58,5 | -60,5 | -71,9 | -61,2 | -66,3 |
| Südamerika | 221.295.874 | 4,0 | 11,7 | 4,8 | -25,7 | -88,6 | -92,4 | -90,8 | -85,0 | -84,1 | -73,6 | -87,6 | -73,5 | -52,3 | -75,1 |
| Südasien | 210.097.623 | 1,7 | 6,6 | 4,4 | -35,0 | -76,7 | -89,9 | -75,1 | -68,1 | -76,9 | -60,6 | -54,7 | -65,5 | -45,7 | -64,0 |
| Golf | 147.994.265 | 2,2 | 9,8 | 1,5 | -45,2 | -84,4 | -90,5 | -86,7 | -75,2 | -84,8 | -79,1 | -71,6 | -79,5 | -76,9 | -77,0 |
| Australien | 87.983.556 | 1,1 | 5,5 | -3,6 | -40,2 | -90,0 | -95,3 | -91,7 | -85,3 | -87,6 | -86,2 | -80,8 | -83,4 | -62,0 | -79,8 |
| Mittlerer Osten | 49.627.031 | 1,4 | 2,3 | -6,1 | -41,8 | -61,9 | -92,2 | -88,0 | -69,7 | -80,2 | -76,7 | -68,4 | -77,5 | -74,2 | -71,0 |
| Nordafrika | 42.008.586 | 9,2 | 12,2 | 4,5 | -43,0 | -86,7 | -94,1 | -93,0 | -77,6 | -85,4 | -82,7 | -77,7 | -84,9 | -76,7 | -79,7 |
| Karibik | 37.348.990 | 5,2 | 2,6 | 4,1 | -36,2 | -81,4 | -92,2 | -86,4 | -66,3 | -77,0 | -69,5 | -77,1 | -78,8 | -51,8 | -70,0 |
| Pazifik | 29.642.710 | 2,5 | 0,9 | -6,5 | -59,6 | -88,7 | -93,1 | -81,1 | -70,9 | -64,9 | -62,3 | -69,3 | -57,8 | -60,0 | -71,0 |
| Ostafrika | 28.917.921 | 6,6 | 15,0 | 10,1 | -30,6 | -71,8 | -90,9 | -87,2 | -73,5 | -80,3 | -75,1 | -52,9 | -74,8 | -68,5 | -69,8 |
| Südliches Afrika | 26.639.673 | 3,8 | 7,1 | -0,6 | -25,8 | -85,8 | -96,1 | -94,8 | -86,3 | -91,4 | -87,2 | -68,7 | -80,1 | -69,2 | -77,2 |
| Zentralamerika | 19.331.963 | 4,9 | 10,6 | 6,9 | -24,3 | -80,3 | -87,7 | -88,6 | -81,5 | -89,5 | -86,6 | -79,6 | -79,7 | -64,9 | -74,9 |
| Westafrika | 18.326.609 | 14,9 | 12,6 | 8,9 | -19,4 | -73,2 | -92,3 | -91,3 | -78,9 | -85,0 | -79,8 | -58,2 | -78,7 | -70,3 | -70,4 |
| Zentralafrika | 9.075.929 | -3,6 | 6,3 | -0,3 | -25,8 | -64,6 | -94,1 | -92,6 | -68,7 | -75,7 | -71,6 | -46,6 | -71,5 | -69,9 | -66,5 |
| Zentralasien | 8.342.277 | 3,1 | 33,5 | 11,0 | -27,4 | -65,6 | -73,6 | -56,9 | -60,6 | -71,9 | -72,4 | -52,1 | -57,9 | -60,5 | -60,8 |

Quelle: Sabre (2021), ab Oktober 2020 vorläufige Daten.

Tabelle 2
**Passagierentwicklung an den deutschen Verkehrsflughäfen im Vorjahresvergleich**

| Periode | Entwicklung im Vergleich zum Vorjahr (in %) |
|---|---|
| 2019 (Gesamtjahr) | +1,5 |
| 2020-1 | -1,6 |
| 2020-2 | -3,7 |
| 2020-3 | -63,0 |
| 2020-4 | -98,6 |
| 2020-5 | -97,9 |
| 2020-6 | -93,7 |
| 2020-7 | -80,1 |
| 2020-8 | -75,6 |
| 2020-9 | -80,7 |
| 2020-10 | -83,2 |
| 2020-11 | -90,3 |
| 2020-12 | -87,9 |
| 2020 insgesamt | -74,6 |

Quelle: ADV (2021).

schaltouristen (ch-aviation, 2020b), gefolgt von weitreichenden, teils behördlich angeordneten Beschränkungen des Verkehrs zwischen China und diversen anderen Staaten ab Ende Januar (ch-aviation, 2020c). Dennoch lag die weltweite Passagierzahl und Verkehrsleistung im Januar 2020 gemäß Daten der Datenbank Sabre Market Intelligence (MI) rund 7,9 % bzw. 7,3 % über den Niveaus des entsprechenden Vorjahresmonats (vgl. Abbildung 1) und damit sogar über dem durchschnittlichen Wachstum des Luftverkehrs von rund 5,3 % (Passagiermenge) bzw. 5,7 % (Passagierkilometer) in den zwölf Monaten zuvor. In den Monaten ab Februar 2020 folgten dann jedoch die bis heute andauernden Rückgänge sowohl der Passagiermenge als auch der Passagierkilometer um bis zu über 80 %. In absoluten Werten entspricht dies einem Rückgang der Zahl der monatlich abfliegenden Passagiere von durchschnittlich 399 Mio. im Zeitraum März bis Dezember 2019 auf nur noch durchschnittlich 116 Mio. im gleichen Zeitraum im Jahr 2020.

Regional fällt der Verkehrsrückgang im Luftverkehr teilweise unterschiedlich aus: dass der durchschnittliche Rückgang im Zeitraum März bis Dezember 2020 in Regionen wie Westeuropa (-79,2 %), der Golf-Region (-77 %), Australien (-79,8 %) oder Nordafrika (-79,7 %) fast 80 % betrug, während die Rückgänge beispielsweise in Fernost (-58,4 %), Osteuropa (-66,3 %) oder Südasien (-64 %) moderater ausfielen. Der Mai 2020 wies in fast allen Regionen den höchsten Rückgang aus. Ebenfalls drastisch war die Entwicklung des Luftverkehrs ab bzw. in Deutschland wie Daten des Flughafenverbandes ADV zeigen (vgl. Tabelle 2): Lag die Zahl der an den deutschen Verkehrsflughäfen (nach Abgrenzung des ADV) gezählten Passagiere im Gesamtjahr 2019 noch leicht (+1,5 %) über Vorjahresniveau, brach sie im zweiten Quartal 2020 um deutlich über 90 % ein. Während insbesondere am Drehkreuz Frankfurt (-96,6 % im Mai 2020) sowie eingeschränkt auch in München und an den größeren Sekundärflughäfen wie Berlin-Tegel, Düsseldorf oder Hamburg eine gewisse Grundversorgung an Flügen aufrecht erhalten werden konnte, ging die Passagierzahl etwa im Mai insbesondere an Regional- und Low Cost-Flughäfen wie Bremen, Dresden, Erfurt, Friedrichshafen, Leipzig/Halle, Münster/Osnabrück, Paderborn, Saarbrücken und Weeze um fast 100 % auf ein zwei- bis dreistelliges Niveau zurück. Eine leichte Erholung im Sommer ist spätestens aufgrund der sogenannten „zweiten Welle“ im Herbst zum Erliegen gekommen.

## Potenzielle Entwicklungspfade des Luftverkehrs und resultierende ökonomische Effekte

Knapp ein Jahr nach Ausbruch der Pandemie ist die Situation von Insolvenzen und erheblichen Streckenstilllegungen der Fluggesellschaften geprägt. Dies hat – zumindest in der kurzen bis mittleren Frist – gravierende ökonomische Auswirkungen auf den Luftverkehrssektor und implizit auf die gesamte Volkswirtschaft. Wie wird sich der internationale Luftverkehr nach der COVID-19-Pandemie in den kommenden Monaten und Jahren weiterentwickeln? Um diese Frage zu beantworten, lohnt zunächst ein Blick in die Vergangenheit. Denn es hat seit den 1970er Jahren immer wieder gravierende Krisen mit Einfluss auf den globalen Luftverkehr gegeben. Hierzu gehören die Ölpreiskrisen, die Anschläge vom 11. September 2001 und die Finanzkrise 2009, um nur einige zu nennen. Wie der Luftverkehr sich daraufhin entwickelt hat, zeigt Abbildung 2. Es fällt auf, dass der Einfluss der Krisen überwiegend kurzfristig war, und dass der globale Luftverkehr in der mittleren und langen Frist regelmäßig zurück zu seinem ursprünglichen Wachstumspfad zurückgekehrt ist. Innerhalb weniger Monate bzw. Jahre wurde das Luftverkehrswachstum in der Vergangenheit wieder maßgeblich durch die Entwicklung des Bruttoinlandsprodukts (BIP) der Länder bzw. Ländergruppen sowie der Einkommen (BIP pro Kopf) getrieben (Scheelhaase, Gelhausen und Maertens, 2020).

Wird der globale Luftverkehr auch nach der gravierenden COVID-19-Pandemie relativ zügig zum ursprünglichen Wachstumspfad zurückkehren? Zu diesem Thema liegen in der Literatur inzwischen einige Prognosen und Szenarien vor. Eine sehr belastbare Prognose wurde von der UN-Organisation ICAO (International Civil Aviation Organization) (2020) in Zusammenarbeit mit IATA Economics und dem IMF erarbeitet. Eine Aktualisierung findet zurzeit monatlich statt. Abbildung 3 zeigt die globalen ICAO-

Abbildung 2
**Entwicklung des weltweiten Luftverkehrs seit 1950**

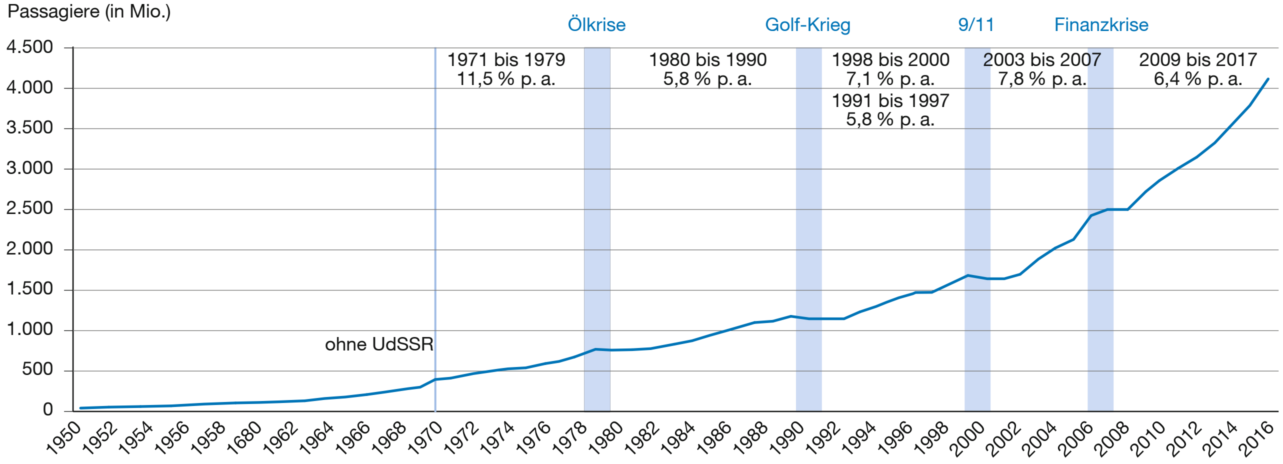

Quelle: Gelhausen, Berster und Wilken (2020).

Szenarien vom 27.1.2021. Weitere Szenarien zur Luftverkehrsentwicklung nach COVID-19 liegen z.B. von EUROCONTROL (2020) vor.

Abbildung 3 zeigt fünf verschiedene Szenarien der globalen Luftverkehrsentwicklung nach der COVID-19-Pandemie (ICAO, 2021), gemessen in Passagierzahlen. Während alle Szenarien grundsätzlich von einer steigenden Nachfrage nach Luftverkehrsdienstleistungen 2021 ausgehen, unterscheiden sie sich hinsichtlich des Verlaufs der jeweils angenommenen Nachfragesteigerung: Szenario 1 ist relativ optimistisch und geht von einer v-förmigen Nachfrageentwicklung aus. Hingegen sind die Annahmen für Szenario 2 etwas pessimistischer, in diesem Szenario wird von einer u-förmigen Entwicklung der Nachfrage ausgegangen. Beide Szenarien haben jeweils ein Sub-Szenario, die eine optimistische bzw. pessimistische Variante abbilden. Weiterhin zeigt Abbildung 3 die empirische Entwicklung der Passagierzahlen 2019 sowie eine – inzwischen überholte – Prognose der Luftverkehrsentwicklung für 2020 und 2021, unter der Annahme, dass die Pandemie nicht eingetreten wäre (Baseline).

Wie aus Abbildung 3 ersichtlich, wird damit gerechnet, dass die Passagierzahlen Mitte 2021 etwa 61 % (Szenario 1) bzw. 53 % (Szenario 2) der ursprünglich, d.h. vor COVID-19 für Mitte 2021 prognostizierten Werte erreichen. Ob und zu welchem Zeitpunkt der internationale Luftverkehr den ursprünglichen Wachstumspfad (Baseline) erreichen kann, hängt ganz wesentlich vom zukünftigen Wirtschaftswachstum in den luftverkehrsrelevanten Weltregionen, insbesondere der Einkommensentwicklung pro Kopf ab. Denn die Entwicklung des BIP sowie des BIP pro Kopf in den jeweiligen Ländern und Ländergruppen sind die wesentlichen Determinanten für die zukünftige Luftverkehrsnachfrage. Politische Maßnahmen auf nationaler und internationaler Ebene können und sollten diese Entwicklung positiv unterstützen.

Erreicht der Luftverkehr mittelfristig nicht wieder das Niveau von 2019, ist mit einem Rückgang der durch den Luftverkehr geschaffenen Bruttowertschöpfung und Beschäftigung zu rechnen. Die Bereitstellung des Luftverkehrsangebots wirkt sich über drei unterschiedliche Effekte auf die Bruttowertschöpfung und Beschäftigung aus: 1. Direkte Effekte entstehen durch die ökonomische

Abbildung 3
**Szenarien 1 und 2: Entwicklung der globalen Passagierzahlen im Vergleich zur Baseline und zu 2019**

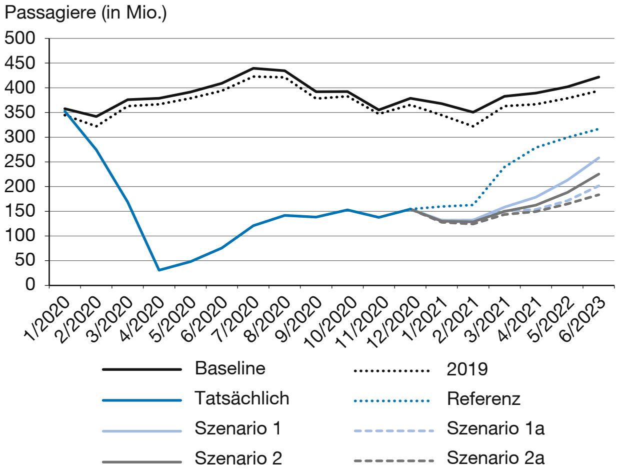

Quelle: International Civil Aviation Organization (ICAO, 2021).

Aktivität der Fluggesellschaften, 2. indirekte Effekte durch die ökonomische Aktivität der Vorleister der Fluggesellschaften (unter anderem Flughäfen, Flugzeughersteller) und 3. induzierte Effekte durch die Verausgabung von Arbeitseinkommen der direkt und indirekt Beschäftigten für Konsumgüter und Dienstleistungen, was wiederum zu ökonomischer Aktivität bei den produzierenden bzw. bereitstellenden Unternehmen führt.

Im Rahmen einer anderen Untersuchung hat das Deutsche Zentrum für Luft- und Raumfahrt (DLR) die direkte, indirekte und induzierte Bruttowertschöpfung sowie Beschäftigung durch den Luftverkehr für Deutschland und die EU28 für 2014 geschätzt (Junior, 2020). Die Berechnung basieren auf der aktuellsten globalen Input-Output-Tabelle der World Input Output Database, die länderübergreifend die Verflechtung der einzelnen Wirtschaftszweige erfasst. Demzufolge stehen in Deutschland etwa 28,9 Mrd. Euro an Bruttowertschöpfung (1,1 % des Bruttoinlandprodukts) und 421.000 Arbeitsplätze im Zusammenhang mit dem Luftverkehr. Bezogen auf die gesamte EU28 ergeben sich 156,9 Mrd. Euro an Bruttowertschöpfung (1,3 % des Bruttoinlandprodukts) und 2,4 Mio. Arbeitsplätze.

Auf der Grundlage dieser DLR-Ergebnisse und der ICAO-Luftverkehrsprognose lassen sich die ökonomischen Auswirkungen der COVID-19-Pandemie für den Luftverkehr grob abschätzen. In der mittleren Frist kann von einem proportionalen Zusammenhang zwischen Verkehrsaufkommen und ökonomischen Effekten ausgegangen werden, da tiefgehende technologische Veränderungen unwahrscheinlich sind und bei konstanten Skalenerträgen die notwendigen Einsatzfaktoren (unter anderem Arbeit) proportional zur Ausbringungsmenge (Verkehrsleistung) steigen bzw. fallen. Die ICAO (2021) prognostiziert für Europa Mitte 2021 einen Rückgang der Passagierzahlen im Vergleich zu 2019 von -42 % (Szenario 1) bzw. -53 % (Szenario 2). Unter der Annahme, dass das Verkehrsaufkommen kurz- bis mittelfristig auf diesem Niveau verbleibt, ergibt sich für Deutschland ein potenzieller Rückgang von 15 Mrd. bis 19 Mrd. Euro an jährlicher Bruttowertschöpfung und 214.000 bis 270.000 Arbeitsplätzen. Für die EU27 und Großbritannien beträgt der mögliche Verlust 86 Mrd. bis 108 Mrd. Euro an jährlicher Bruttowertschöpfung und 1,3 Mio. bis 1,6 Mio. Arbeitsplätze.

### Ordnungspolitische COVID-19-Eingriffe verursachten negative ökonomische Effekte im Luftverkehr

Die durch die COVID-19-Pandemie verursachten negativen ökonomischen Effekte sind im Luftverkehrssektor besonders stark. Während die Nachfrage nach Konsumgütern relativ schnell das Vorkrisenniveau erreichte, ist die Nachfrage nach Dienstleistungen und im Besonderen im Luftverkehr noch immer (OECD, 2020a, Stand: Februar 2021) drastisch reduziert. Flughäfen erwarten weltweit für 2020 einen Umsatzverlust von ca. 104 Mrd. US-$, bei einem Vorjahresumsatz von ca. 170 Mrd. US-$ (ACI, 2020). Fluggesellschaften gehen weltweit für 2020 von ca. 400 Mrd. US-$ Umsatzverlust aus, bei einem Vorjahresumsatz von ca. 880 Mrd. US-$. Ein hoher Fixkostenanteil erschwert es Fluggesellschaften und Flughäfen zudem, flexibel auf den Nachfrageschock zu reagieren, weshalb mittelfristig hohe branchenweite Verluste erwartet werden (IATA, 2020a; IFC, 2020).

Diese Zahlen verdeutlichen, dass es ohne staatliche Unterstützung zu weitreichenden strukturellen Anpassungen im Luftverkehrssektor kommt. Der Ausgang ist dabei offen. Die meisten Regierungen sehen den Luftverkehr als einen strategischen Sektor an, der für das gesamtökonomische Wachstum entscheidend ist (Zhang und Graham, 2020). Politische Entscheidungen wurden hierdurch in der Vergangenheit beeinflusst, auch weil Staaten häufig relevante Anteilseigener bei Fluggesellschaften und Flughäfen sind. Dies hat die Schaffung von regionalen und nationalen Oligopolen im Luftverkehr begünstigt und eine Pfadabhängigkeit erschaffen. Einzelne Unternehmen haben eine systemische Relevanz erlangt, ohne die eine funktionierende Infrastruktur flächendeckend mittelfristig gefährdet ist. Bei den meisten Flughäfen der Welt sind öffentliche Träger die größten Anteilseigner. Theoretisch stehen somit ausreichend Mittel zur Verfügung, um die Verluste der COVID-19-Krise an den Flughäfen auszugleichen. Der Fokus fällt deshalb auf die Fluggesellschaften, bei denen Staaten seltener und in einem geringeren Umfang beteiligt sind. Die verfügbaren liquiden Mittel und damit ihre Widerstandsfähigkeit unterscheiden sich relativ stark zwischen den Fluggesellschaften, wodurch viele die Krise mit eigenen Mitteln nicht überstehen werden (IATA, 2020b). Hierbei ist zu berücksichtigen, dass staatliche Corona-Hilfen etablierte Strukturen weiter verfestigen könnten, weil große Unternehmen in der Regel erfolgreicher im Rentseeking sind (Abate, Christidis und Purwanto, 2020).

Die durch die Pandemie verursachte ökonomische Krise unterscheidet sich in ihrer Verantwortung von vorherigen Krisen, da politische Maßnahmen zur Pandemiebekämpfung ursächlich sind. Dies verändert die ordnungspolitischen Bewertungskriterien wirtschaftspolitischer Maßnahmen, sofern sie Schäden kompensieren, die durch das vorrangige Ziel des Infektionsschutzes entstanden sind. Viele Staaten haben deshalb gesamtwirtschaftliche Maßnahmen ergriffen und z. B. den Umfang von Arbeitslosen- oder Kurzarbeitergeld erweitert, Steuern und Abgaben gesenkt oder deren Zahlungsfristen verschoben. Grundsätzlich haben diese Maßnahmen einen wichtigen,

Tabelle 3
**Strukturierte Darstellung unterschiedlicher Finanzierungsmöglichkeiten**

| Instrument | Einzelwirtschaftliche Sicht | Gesamtwirtschaftliche Sicht |
|---|---|---|
| Kein staatlicher Eingriff | - Kein Staatseinfluss<br>- Gefahr der Insolvenz oder Überschuldung<br>- Notwendigkeit der Beschaffung von Kapital am Kapitalmarkt | - Kein staatlicher Kapitalbedarf<br>- Keine Wettbewerbsverzerrung<br>- Kein Einfluss auf Branchenentwicklung, Mobilitätsangebot und Arbeitsplätze |
| Muddling Through | - Nur kurzfristiger Staatseinfluss<br>- Gefahr der Insolvenz oder Überschuldung<br>- Notwendigkeit der Beschaffung von Kapital am Kapitalmarkt | - moderater öffentlicher Kapitalbedarf<br>- keine Wettbewerbsverzerrung<br>- wenig Einfluss auf Branchenentwicklung und Mobilitätsangebot<br>- kurzfristige Sicherung von Arbeitsplätzen durch Kurzarbeit |
| Staatlich abgesicherter Kredit | - Geringer Staatseinfluss<br>- Fremdkapitalbeschaffung wird erleichtert, geringere Zinsen<br>- Gefahr der Überschuldung | - Kein staatlicher Kapitalbedarf<br>- Übernahme des Kreditrisikos<br>- Geringe Gefahr der Wettbewerbsverzerrung |
| Staatlicher Kredit | - Geringer Staatseinfluss<br>- Fremdkapitalbeschaffung wird gesichert, geringere Zinsen<br>- Gefahr der Überschuldung | - Staatlicher Kapitalbedarf<br>- Übernahme des Kreditrisikos<br>- Geringe Gefahr der Wettbewerbsverzerrung |
| Nicht rückzahlbarer Zuschuss | - Geringer Staateinfluss<br>- Eigenkapital wird erhöht | - Staatlicher Kapitalbedarf<br>- Erhöhung der Staatsverschuldung<br>- Hohe Gefahr der Wettbewerbsverzerrung |
| Staatsbeteiligung | - Hoher Staatseinfluss<br>- Eigenkapital wird erhöht<br>- Verwässerung der Anteile der Aktionäre | - Staatlicher Kapitalbedarf<br>- Hohe Gefahr der Wettbewerbsverzerrung<br>- Einfluss auf Branchenentwicklung, Mobilitätsangebot und Arbeitsplätze über Aufsichtsrat |
| Kombinationen | - Je nach genutzten Maßnahmen obige Vor- und Nachteile | - Je nach genutzten Maßnahmen obige Vor- und Nachteile |

Quelle: eigene Zusammenstellung.

aber begrenzten Effekt, der nicht ausreichen dürfte, um die Folgen des COVID-19 bedingten Konjunktureinbruchs im Luftverkehrssektor abzufedern (Devereux et al., 2020). Hinzu kommt, dass durch die gesamtwirtschaftlichen Maßnahmen vor allem die Nachfrageseite stimuliert wird, diese aber im Luftverkehrssektor auf Mobilitätsbeschränkungen trifft. Ordnungspolitisch betrachtet sind staatliche COVID-19-Hilfen im Luftverkehrssektor vor allem dann sinnvoll, wenn sie darauf ausgerichtet sind, die durch die Eindämmungsmaßnahmen verursachten zusätzlichen Wettbewerbsverzerrungen zu reduzieren, ohne neue zu schaffen. Aufgrund der besonderen Betroffenheit und den speziellen Eigenschaften des Luftverkehrs sind sektorspezifische Maßnahmen aus ordnungspolitischer Sicht effizient, da hierdurch die Zahl der Unternehmen aus weniger betroffenen Sektoren minimiert wird, welche die COVID-19-Hilfen als Trittbrett nutzen, um strukturelle Defizite auszugleichen.

In der internationalen Politik scheint Einigkeit darüber zu herrschen, dass der Luftverkehr eine besondere Stellung in der COVID-19-Krise einnimmt. Wenn Länder sektor- oder firmenspezifische Maßnahmen ergreifen, dann betreffen sie vor allem den Luftverkehr (OECD, 2020b). Laut IATA (2020b), dem größten Interessensverband der Airlines, haben Fluggesellschaften weltweit bis Anfang September 2020 mindestens 161 Mrd. US-$ staatliche Unterstützung erhalten. Der Großteil bestand aus Zuschüssen oder Krediten. Vergleichbare Hilfszahlungen für Flughäfen sind nicht erfolgt.

Wenn nicht alle Fluggesellschaften gleichermaßen staatliche Hilfen erhalten, besteht die Gefahr, dass nicht die produktivsten Fluggesellschaften die Krise überstehen, sondern jene, die weniger durch Maßnahmen der Pandemieeindämmung betroffen sind oder ohnehin mehr staatliche Unterstützung erhalten. Durch Insolvenzen und Zusammenschlüsse könnte es durch weitere Oligopol- und Monopolbildungen zu Preissteigerungen kommen. Bereits jetzt werden 36 % aller Routen nur von zwei bis drei Fluggesellschaften durchgeführt (OECD, 2020b). An dieser Stelle offenbart sich das Dilemma, vor dem die Politik steht. Sowohl mit als auch ohne staatliche Rettungsaktionen besteht die Gefahr einer zunehmenden Unternehmenskonzentration, die den Wettbewerb hemmt.

Hinzu kommt, dass der akute Pandemieverlauf einen direkten Einfluss auf die potenziellen Maßnahmen hat. Dauert die Pandemie an, ist eine reduzierte Mobilität der Bevölkerung immer noch ein vorrangiges politisches Ziel, weshalb z. B. die Stimulierung der Nachfrageseite nicht sinnvoll ist (Devereux et al., 2020). Hierdurch ergibt sich die Frage, welche konkreten Maßnahmen überhaupt geeignet sind, um die Interessen aller Stakeholder zu berücksichtigen.

Eine strukturierte Abbildung typischer Maßnahmen im Luftverkehrssektor kann Tabelle 3 entnommen werden. Staatliche Kredite oder staatlich abgesicherte Kredite sind aktuell die am häufigsten genutzte Form der Staatshilfe im Luftverkehrssektor. Diese werden häufig, wie am Beispiel der Lufthansa oder Air France zu sehen ist, firmenspezifisch vergeben, wodurch die Gefahr der Diskriminierung und Wettbewerbsverzerrung gegenüber produktiveren nationalen Mitbewerbern besteht. Die Gefahr der Diskriminierung ließe sich verhindern, indem Kredite sektorweit gewährleistet, an transparente Bedingungen geknüpft und zu gleichen Konditionen vergeben werden. Der maximale Kreditrahmen kann durch Kennzahlen wie z. B. dem Umfang der Betriebsmittel berechnet werden. Um einer Wettbewerbsverzerrung entgegenzutreten, können Unternehmen – wie im Fall der Lufthansa – gezwungen werden, begehrte Flughafenslots an Mitbewerber abzugeben oder die Flotte zu verkleinern (Abate, Christidis und Purwanto, 2020). Der staatlich abgesicherte Kredit hat im Gegensatz zum staatlichen Kredit den Vorteil, dass der staatliche Kapitalbedarf erst bei einem Zahlungsausfall des Unternehmens entsteht und die Kosten der Kapitalbeschaffung für den Staat somit geringer sind. Andererseits entsprechen die Kreditkonditionen abgesicherter Kredite eher gängigen Marktbedingungen, wodurch die Zinslast für die Unternehmen steigt. Als Gegenleistung für günstige Konditionen können Staaten Kredite – wie im Fall der vereinbarten $CO_2$-Reduzierung der Air France – effektiv nutzen, um politische Ziele durchzusetzen, indem der Zinssatz an das Erreichen von Zielvorgaben gekoppelt wird (Hepburn et al., 2020). In Deutschland werden Hilfskredite bisher nicht an politische Zielvorgaben geknüpft, auch wenn sie – wie etwa eine $CO_2$-Reduzierung – dem grundsätzlichen politischen Willen entsprechen. Dennoch kann es politisch opportun sein, Einflussnahme auf strategische Unternehmensentscheidungen zu vermeiden. In der COVID-19-Krise besteht eine Gefahr auch darin, dass sich der Markt durch wandelnde Konsumentenpräferenzen besonders schnell verändert und der Staat durch Zielvorgaben notwendige strukturelle Veränderungen im Unternehmen bremst und hierdurch dem Unternehmen langfristig schadet (OECD, 2020b).

Sowohl beim staatlichen als auch beim staatlich abgesicherten Kredit erhöhen die vergünstigten Zinskonditionen und die großzügigen Kreditrahmen die Gefahr einer Überschuldung. Die Gefahr der Überschuldung ist bei nichtrückzahlbaren Zuschüssen nicht gegeben, da sie das Eigenkapital von Unternehmen erhöhen. Zuschüsse werden in der Regel an keine Bedingungen gekoppelt, können jedoch zielgerichtet sein. In Italien oder Brasilien werden z. B. fällige Entschädigungszahlungen durch ausgefallene Flüge mithilfe öffentlicher Finanzmittel finanziert. In den USA werden durch den CARES Act die Gehaltszahlungen im Luftverkehrssektor vom Staat getragen. Auf diese Weise können zum einen politische Ziele wie die Vermeidung von Arbeitslosigkeit oder Verbraucherschutz gewährleistet werden, und zum anderen vergrößert das höhere Eigenkapital den Handlungsspielraum der Unternehmen, wodurch diese in die Lage versetzt werden, mithilfe eigener Lösungen auf die Krise zu reagieren. Nicht rückzahlbare Zuschüsse sollten möglichst sektorübergreifend zu einheitlichen Konditionen zugänglich sein, da sie ansonsten zu einer starken Wettbewerbsverzerrung führen können (OECD, 2020b). Ob ein solcher Zuschuss aus ordnungspolitischer Sicht zu rechtfertigen ist, muss sorgfältig geprüft werden, da er die Staatsverschuldung direkt erhöht. Durch mobilitätsbeschränkende Eindämmungsmaßnahmen gibt es jedoch eine politische Verantwortung, und je länger die COVID-19-Krise andauert, desto sinnvoller werden nicht rückzahlbare Zuschüsse, um eine Überschuldung im Luftverkehr zu vermeiden.

Für eine Abfederung der durch die Corona-Pandemie verursachten dramatischen negativen ökonomischen Effekte im Luftverkehr ist eine Kombination von Entlastungen und Finanzierungshilfen erforderlich. Um Diskriminierung und Wettbewerbsverzerrung zu vermeiden, sollten Hilfsmaßnahmen sektorweit zu den gleichen Konditionen zugänglich sein und in Einklang mit anderen politischen Zielvorgaben gebracht werden. Hinzu kommt, dass mit einer andauernden Krise die Überschuldung ein immer größeres Problem wird, wodurch der Einsatz von zielgerichteten nicht rückzahlbaren Zuschüssen sinnvoll ist.

## Schlussfolgerungen und Empfehlungen

Dieser Aufsatz verdeutlicht, dass die COVID-19-Pandemie eine existenzbedrohende ökonomische Krise im Luftverkehrssektor ausgelöst hat. Durch weltweite Eindämmungsmaßnahmen des Infektionsschutzes ist es durch Angebotsbeschränkungen und Nachfrageeinbrüche zu einem außergewöhnlich starken Rückgang des Passagierluftverkehrs gekommen, der regional sehr heterogen ausgefallen ist. Vor allem Nord- und Südamerika sowie Westeuropa und Südostasien sind mit Rückgängen von bis zu 90 % besonders betroffen. Dies gilt auch für den deutschen Luftverkehr mit Rückgängen von bis zu 98 %.

Die ökonomische Tragweite der Krise ist im Luftverkehr noch nicht vollständig abzuschätzen. Erreicht der Luftverkehr in den kommenden Jahren nicht wieder das Niveau von 2019, ist mit einem Rückgang der durch den Luftverkehr geschaffenen Bruttowertschöpfung und Beschäftigung zu rechnen. Zu welchem Zeitpunkt der Luftverkehr den ursprünglichen Wachstumspfad erreichen kann, hängt wesentlich vom Infektionsgeschehen und dem zukünftigen Wirtschaftswachstum in den luftverkehrsrelevanten Weltregionen ab. Vorherige Krisen des Luftverkehrs zeigen, dass diese überwiegend kurzfristige Effekte hatten und dass der globale Luftverkehr in der mittleren und langen Frist regelmäßig zurück zu seinem ursprünglichen Wachstumspfad zurückgekehrt ist. Politische Maßnahmen auf nationaler und internationaler Ebene können und sollten diese Entwicklung positiv unterstützen, um eine möglichst rasche Erholung zu ermöglichen.

Aufgrund der besonderen Betroffenheit des Luftverkehrssektors sind gesamtwirtschaftliche Maßnahmen wie die Erweiterung des Kurzarbeitergeldes oder Steuersenkungen aber nicht ausreichend und sollten um sektorspezifische Maßnahmen erweitert werden. Dies ist in vielen Staaten erfolgt: Staatliche Kredite oder staatlich abgesicherte Kredite sind aktuell die am häufigsten genutzte Form der Staatshilfe im Luftverkehrssektor. Diese ermöglichen einen ausgewogenen Trade-off zwischen staatlicher Einflussnahme, Staatsverschuldung, Wettbewerbsverzerrung, sofern sie an transparente und universelle Konditionen gekoppelt sind. Je länger die Krise jedoch andauert, desto kritischer wird die Überschuldung von Unternehmen. Nicht rückzahlbare Zuschüsse bieten in diesem Fall eine einfache, aber kostspielige Lösung, da sie die Staatsverschuldung direkt erhöhen. Grundsätzlich muss natürlich auch die Dynamik des Infektionsgeschehens bei der Gestaltung von Hilfsmaßnahmen einbezogen werden.
